# Supplementary material for: Mechanisms governing the pioneering and redistribution capabilities of the non-classical pioneer PU.1
Source: Nat Commun. 2020 Jan 21;11:402. doi: 10.1038/s41467-019-13960-2 (PMC6972792; doi:10.1038/s41467-019-13960-2)
Supplement: Supplementary file 7 — Source data [file 41467_2019_13960_MOESM7_ESM.zip › Source_Data/FigureS2/FigureS2B_MotifScanOutput/KmeansCluster3/homerResults/motif6.similar.html]

motif6

## Information for motif6

T
A
G
C
C
T
G
A
A
G
T
C
A
C
G
T
A
C
G
T
T
A
G
C
G
C
A
T
T
C
A
G
G
A
T
C
G
C
T
A
  
Reverse Opposite:  

C
G
A
T
C
T
A
G
A
G
T
C
C
G
T
A
A
T
C
G
C
G
T
A
C
G
T
A
T
C
A
G
G
A
C
T
A
T
C
G
  

|  |  |
| --- | --- |
| p-value: | 1e-225 |
| log p-value: | -5.195e+02 |
| Information Content per bp: | 1.686 |
| Number of Target Sequences with motif | 938.0 |
| Percentage of Target Sequences with motif | 19.67% |
| Number of Background Sequences with motif | 2218.6 |
| Percentage of Background Sequences with motif | 5.23% |
| Average Position of motif in Targets | 99.5 +/- 50.3bp |
| Average Position of motif in Background | 99.2 +/- 73.7bp |
| Strand Bias (log2 ratio + to - strand density) | -0.1 |
| Multiplicity (# of sites on avg that occur together) | 1.04 |
| Motif File: | file (matrix) reverse opposite |

### Similar de novo motifs found

|  |  |  |  |  |  |  |  |
| --- | --- | --- | --- | --- | --- | --- | --- |
| Rank | Match Score | Redundant Motif | P-value | log P-value | % of Targets | % of Background | Motif file |
| 1 | 0.850 | T C G A T C G A C T G A C G T A C T A G A T G C C G T A A C T G C G T A C G T A T A C G C G A T A T C G T G A C | 1e-142 | -327.710750 | 4.36% | 0.18% | motif file (matrix) |
| 2 | 0.811 | T A G C T C G A A T G C C G A T A C G T A G T C A C G T T A C G G A T C C G A T A G C T A G C T A G C T | 1e-135 | -311.105994 | 5.24% | 0.42% | motif file (matrix) |
| 3 | 0.860 | T C G A C G T A C T G A A G T C C G T A C T A G G T C A G T C A T A C G G A C T T C A G | 1e-92 | -213.280883 | 12.92% | 4.76% | motif file (matrix) |
| 4 | 0.871 | T C G A T C G A G C T A C A T G A T G C C G T A A T C G G T C A C G T A T A C G C G A T A T C G | 1e-88 | -202.964375 | 2.83% | 0.14% | motif file (matrix) |
| 5 | 0.798 | C T A G A G T C C G T A A C T G G T C A C G T A A C T G | 1e-32 | -75.095841 | 28.01% | 20.31% | motif file (matrix) |
